# Effect of Mono- and Multichlorinated Organic Compounds—Chlorocyclohexane and Hexachloro-*p*-xylene—On the Catalytic Properties of Titanium–Magnesium Catalysts in the Homo- and Copolymerization of Ethylene with 1-Hexene

**DOI:** 10.3390/ijms231810335

**Published:** 2022-09-07

**Authors:** Ildar I. Salakhov, Tatiana B. Mikenas, Vladimir A. Zakharov, Valeriy G. Kozlov, Mikhail A. Matsko, Tatiana N. Suslova

**Affiliations:** 1 R&D Center PJSC Nizhnekamskneftekhim, 423574 Nizhnekamsk, Russia; 2 Boreskov Institute of Catalysis, Siberian Branch of the Russian Academy of Sciences, 630090 Novosibirsk, Russia; 3 Institute of Ecology of the Volga River Basin, Russian Academy of Sciences, 445003 Togliatti, Russia

**Keywords:** titanium-magnesium catalyst, chlorinated organic compound, ethylene, 1-hexene copolymerization

## Abstract

Ethylene polymerization and ethylene/1-hexene copolymerization over the titanium–magnesium catalytic system in the presence of chlorocyclohexane (CHC) and hexachloro-p-xylene (HCPX) has been studied. Modification of TMC with chlorocyclohexane and hexachloro-p-xylene increased catalyst activity severalfold for both ethylene polymerization and ethylene/1-hexene copolymerization. The key kinetic regularities of ethylene homopolymerization and ethylene/1-hexene copolymerization in the presence of CHC and HCPX were determined, and the copolymerization constants were measured. Molecular characteristics and the copolymer composition were determined for the synthesized samples of ethylene homopolymers and ethylene/hexene copolymers. Modification of the titanium–magnesium catalyst with chlorinated organic compounds reduced 1-hexene content in the copolymer; polymerization was sensitive to 1-hexene as a regulator of polymer molecular weight. The potential mode of action of chlorinated organic modifiers on catalytic properties of titanium–magnesium catalyst is discussed.

## 1. Introduction

Polyethylene (PE) is one of the largest-scale polymers [1]. The annual world production volume of polyethylene of different brands is more than 100 million tons, making up to 40% of the total production volume of thermoplastic materials [2].

Titanium–magnesium Ziegler–Natta catalysts (TMCs) are predominantly used when producing PE using the slurry-phase and gas-phase polymerization technologies [1,2,3]. The application of TMCs is dictated by the high activity and stability of catalytic systems, ample opportunities for regulating the molecular characteristics of the resulting polyethylene, and the potential of producing ethylene/α-olefin copolymers. Today, there exist two main trends in the improvement of TMCs: optimizing the methods used for catalyst preparation and modifying the catalytic systems by introducing various additives (promoters) [4,5,6,7,8].

Until the 2000s, activation with chlorinated organic compounds was performed mainly for “vanadium” catalysts [9,10,11,12,13]. Recently, an appreciably large number of studies focusing on modification of titanium–magnesium catalysts with chlorinated organic compounds (COCs) have been reported [4,5,14,15,16,17,18,19,20,21]. The impact of a wide range of different compounds has been evaluated. Thus, patents [15,22,23,24,25] focus on trichlorofluoromethane (CFCl_3_), chloroethane (C_2_H_5_Cl), dichloroethane (C_2_H_4_Cl_2_), tetrachloromethane (CCl_4_), tetrabromomethane (CBr_4_), dichloromethane (CH_2_Cl_2_), 1,1,1-trichloroethane (CH_3_CCl_3_), and chloroform (CHCl_3_), which promote olefin polymerization. Halogenated hydrocarbons have been shown to increase the yield of polymers, reduce the rate of ethane formation, and allow the regulation of molecular weight of PE.

The effect of the presence of monochlorinated organic compounds with the number of carbon atoms ranging from 2 to 6 (chloroethylene [19], chlorobutyl [16,17], chlorocyclopentane [4], and chlorocyclohexane [4,5,16,18,20,21]) on PE polymerization was studied. Among monochlorinated organic compounds, chlorocyclohexane (CHC) exhibits the strongest promoting activity [4,5,16,18,20].

The activity of TMC decreases in a series of chlorinated hydrocarbons: acyclic > aliphatic > aromatic compounds [4,16]. The use of alkenyl halides causes catalyst poisoning. It has been found that, among acyclic compounds, the presence of chlorocyclohexane ensures the best results (better polymer yield, size distribution of PE powder particles (the morphology of polymer powder), higher degree of crystallinity and, therefore, greater melt flow index (MFI) for the resulting polyethylene). Chlorocyclohexane provides highly efficient modification of TMCs containing internal donors based on alcohol and ether moieties [18,20]. Therefore, CHC may be an efficient agent for modifying titanium–magnesium Ziegler–Natta catalysts.

However, in refs. [4,5,14,16,18,20], focus has been on the use of CHC during ethylene homopolymerization, and almost no data are available on the effect of COCs on ethylene/α-olefin copolymerization. Most of the food-grade PE brands produced in the world are ethylene/α-olefin copolymers. Therefore, it is important from both fundamental and practical perspectives to study ethylene/α-olefin copolymerization over TMCs modified with chlorinated organic compounds. Comparison of a series of α-olefins (e.g., for producing the bimodal high-density PE (HDPE)) revealed that better mechanical properties are achieved for the polymers produced upon copolymerization with 1-hexene than with 1-butene [26], while some characteristics of the film prepared from linear low-density polyethylene (LLDPE) are improved when 1-hexene and 1-octene are used during polymerization [27].

A monochlorinated cyclic compound, CHC, and a multichlorinated aromatic compound, hexachloro-p-xylene (HCPX), have been used as promoters for TMC. HCPX is a transparent crystalline powder that is used for manufacturing industrial rubber goods and drugs. As it has been observed previously [28,29,30], HCPX is highly efficient for modifying Ziegler–Natta catalysts (butadiene polymerization over a homogeneous neodymium catalyst [28] and ethylene/propylene copolymerization over a vanadium-based catalyst [29]).

The effect of chlorocyclohexane and hexachloro-p-xylene on ethylene polymerization and ethylene/1-hexene copolymerization over titanium–magnesium catalysts has been addressed. The properties of the resulting polymers suggest features of the mode of action of the promoters used in the catalytic systems.

## 2. Results and Discussion

### 2.1. Effect of the Nature of Chlorinated Organic Compounds on Catalytic Activity of TMC

#### 2.1.1. Ethylene Homopolymerization in the Presence of CHC and HCPX

Two chlorinated organic compounds (COCs), monochlorocyclohexane and hexachloro-p-xylene, were used as promoters for the titanium–magnesium catalyst for ethylene polymerization. With the optimal content of chlorinated organic compounds in the catalytic system determined preliminarily, it was found that the maximum catalytic activity was observed for both CHC and HCPX at the COC/Ti molar ratio = 1. Therefore, the equimolar Ti/COC ratio was used to further investigate the effect of chlorinated organic compounds on ethylene polymerization and ethylene/1-hexene copolymerization, as well as properties of high-molecular-weight compounds produced over Ti–Mg catalysts.

**Figure 1:**
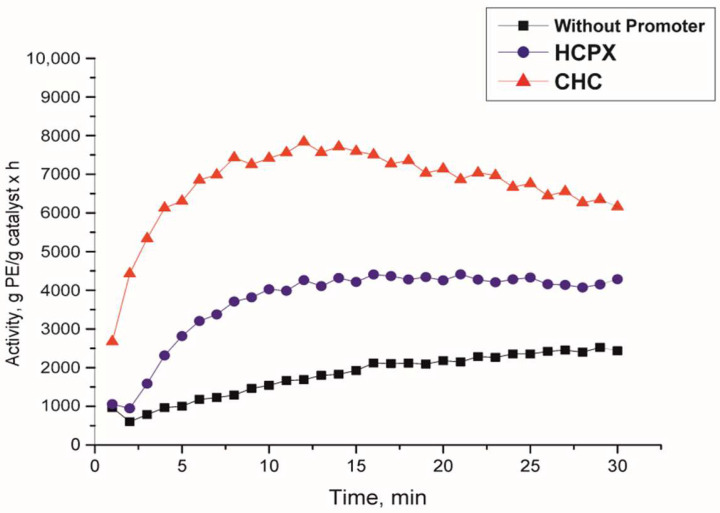
Effect of the presence of COC on the curve showing the dependence between activity of the catalytic system and duration of ethylene polymerization (the polymerization conditions are listed in Table 1): exp. 1—non-modified TMC, exp. 4—TMC modified with CHC, (C_6_H_11_Cl)/Ti = 1 (mol/mol), exp. 7—TMC modified with HCPX, (C_6_H_4_ (CCl_3_)_2_)/Ti = 1 (mol/mol).

Table 1 and Figure 1 show the data characterizing the activity of the titanium–magnesium catalytic system (*W_G_*, *W_Ti_*) in ethylene polymerization and the effect of the chemical nature of COC on the activity of the catalytic system. The addition of CHC and HCPX to the polymerization system increases the activity of the catalytic system (Table 1, exp. 1, 4, and 7).

The yield of polyethylene per unit mass of the initial catalyst and, therefore, per unit mass of titanium, increased most significantly when CHC was added to the system (Table 1, exp. 4). The activating ability of HCPX was somewhat lower than that of CHC under these conditions (Table 1, exp. 7).

A higher polymer yield (i.e., the retained higher activity of TMC after addition of both CHC and HCPX) was observed during the entire polymerization process (Figure 1).

**Figure 2:**
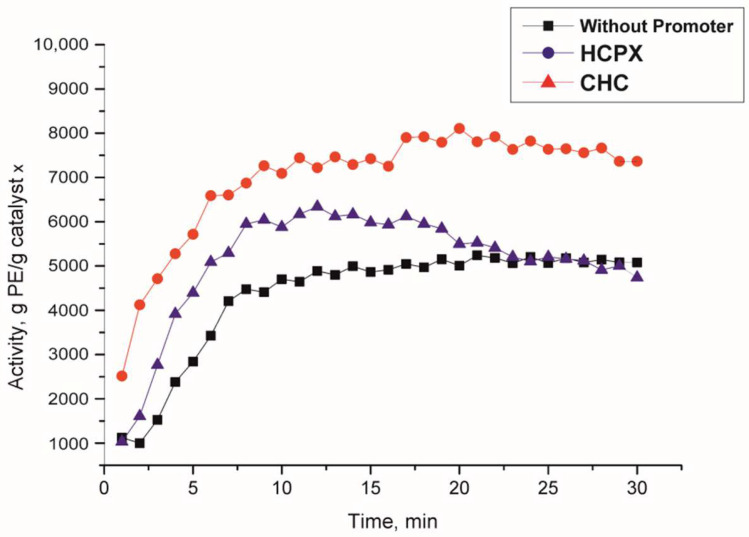
Activity of the catalytic system in ethylene/1-hexene copolymerization as a function of process duration. [1-hexene] = 0.16 mol/L. 2—TMC; 8—TMC–HCPX; 5—TMC–CHC. The conditions are listed in Table 1.

In this case, the promoting role played by the analyzed COCs on the Ziegler–Natta TMC is obvious. In the absence of any COC, the yield of the polymer consistently increased rather uniformly during 30 min of polymerization. In the presence of COC, regardless of its chemical nature, the yield of polyethylene increased most intensely during the initial 15 min of polymerization. During the next 15 min of the process, the yield of the polymer and activity of the catalytic system containing HCPX remained virtually unchanged, while the catalytic system containing CHC was even characterized by certain decline in activity (Figure 1, exp. 4 and 7). Therefore, the non-modified TMC attained activity of 2 kg PE/g cat·h within 30 min of polymerization under experimental conditions (Figure 2, exp. 1). After 15 min, the TMC–HCPX system reached an activity of 2 kg PE/g cat·h (Figure 2, exp. 7). Unlike the two systems discussed above, the TMC modified with CHC reached the maximum activity of 7.5–7.7 kg PE/g cat·h within 13–15 min; its activity then gradually decreased to 6.1 kg PE/g cat·h (Figure 1, exp. 4).

The catalyst color changed after it had been treated with the COC. While initially being grayish brown, the catalyst slurry in heptane became brown after treatment with HCPX or dark beige after treatment with CHC. This fact indicates that chemical interaction (and possibly complexation) took place in the TMC–COC system.

#### 2.1.2. Ethylene/1-Hexene Copolymerization in the Presence of CHC and HCPX

An analysis of the recorded copolymerization patterns shows that the addition of an α-olefin noticeably increased the yield of ethylene/1-hexene copolymer per unit mass of catalyst and titanium in the catalytic system compared to the yield of the homopolymer (Table 2, exp. 1–3). Within the studied limits of 1-hexene content in the system, catalyst activity increased most significantly for α-olefin concentration of 0.16 mol/L. The yield of the polymer increased virtually 2.4-fold (Table 2, exp. 1, 2). Figure 1 and Figure 2 suggest that not only did the efficiency of the catalytic system increase, but the maximum catalyst activity was attained much sooner than during homopolymerization.

The activity of the TMC modified with COC upon copolymerization (Table 2, exp. 2 and 8; Figure 2, exp. 5 and 8) increased less significantly than it did upon ethylene homopolymerization. Thus, after 20 min of the experiment, activities of the non-modified TMC and TMC modified with HCPX became almost identical (Figure 2, exp. 2 and 8).

TMC modified with CHC exhibited the highest activity in ethylene/1-hexene copolymerization, as it was for ethylene homopolymerization (Table 2, exp. 4; Figure 2, exp. 5), but in the presence of CHC, adding α-olefin to the system, regardless of its concentration, did not result in catalyst promotion (Table 2).

**Figure 3:**
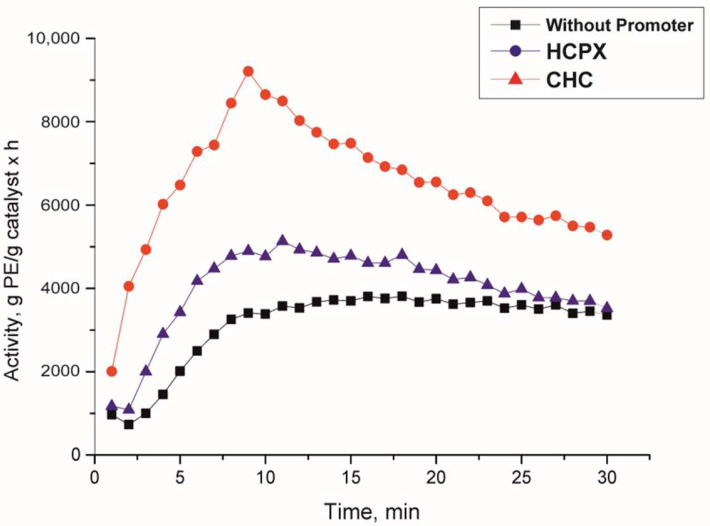
Activity of the catalytic system upon ethylene/1-hexene copolymerization as a function of process duration. [1-hexene] = 0.32 mol/L. 3—TMC; 9—TMC–HCPX; 6—TMC–CHC. The conditions are listed in Table 1.

As 1-hexene concentration rose from 0.16 to 0.32 mol/L, catalyst efficiency in copolymerization decreased regardless of the nature of COC and whether it was present in the system or not (Table 1; Figure 2 and Figure 3). The shape of the curve showing the dependence of activity of the catalytic system on process duration remained almost unchanged for copolymerization over the non-modified TMC and TMC/HCPX.

In general, the curves showing the dependence of activity on polymerization duration for the analyzed catalyst have a typical shape (like that for titanium-magnesium catalysts). At the initial polymerization time, monomer consumption increases and the reaction rate declines after a certain instant of time.

For the TMC/CHC catalytic system, identically to the case of ethylene homopolymerization, there was a maximum in catalyst activity after 10 min of polymerization; i.e., the process was non-steady-state. As a result, catalyst activity declined from 9.2 to 5.3 kg/g cat·h after 30 min of the experiment (Figure 3, exp. 6).

Table 3 summarizes the data on the content of hexene monomers in ethylene/1-hexene copolymers produced at different comonomer concentrations in the polymerization medium in the presence of TMC (non-modified TMC or TMC modified with COC). The copolymers synthesized using CHC and HCPX in the catalytic system contained fewer hexene monomers than those produced over the TMC without a promoter. The density values of the samples proportionally decreased with an increase in the content of 1-hexene in PE, depending on the synthesis conditions with different COCs.

The data on copolymerization constants (Table 4) demonstrate that this monomer ratio was quite predictable. The constant r_1_ was significantly higher than unity and several orders of magnitude higher than r_2_, which was lower than unity in all cases. This fact demonstrates unambiguously that ethylene is attached to both types of active sites more readily than 1-hexene.

The presence of COCs increased the chances for ethylene to be incorporated into the polymer chain, while the probability of 1-hexene attachment declines, especially in the case of HCPX. The product of copolymerization constants remains nearly equal to unity, being indicative of statistical distribution of comonomers in the polymer chain.

Hence, the modification of TMC with chlorinated organic compounds both increased the catalyst activity and reduced the probability of 1-hexene incorporation into the macromolecule, especially when HCPX was used.

### 2.2. The Effect of TMC Modification with Chlorinated Organic Compounds on Polyethylene Properties

#### 2.2.1. Molecular Characteristics and the MFI of the Polymers

The MFI values were determined for the polymer samples produced in the presence of the TMC/TIBA catalytic system modified with different chlorinated organic compounds. The melt flow index depends on the weight average molecular weight (*M*_w_) of polyolefins and is one of the rheological parameters used most frequently. The MFI increases with rising molecular weight, while melt flowability increases as *M*_w_ is reduced.

**Figure 4:**
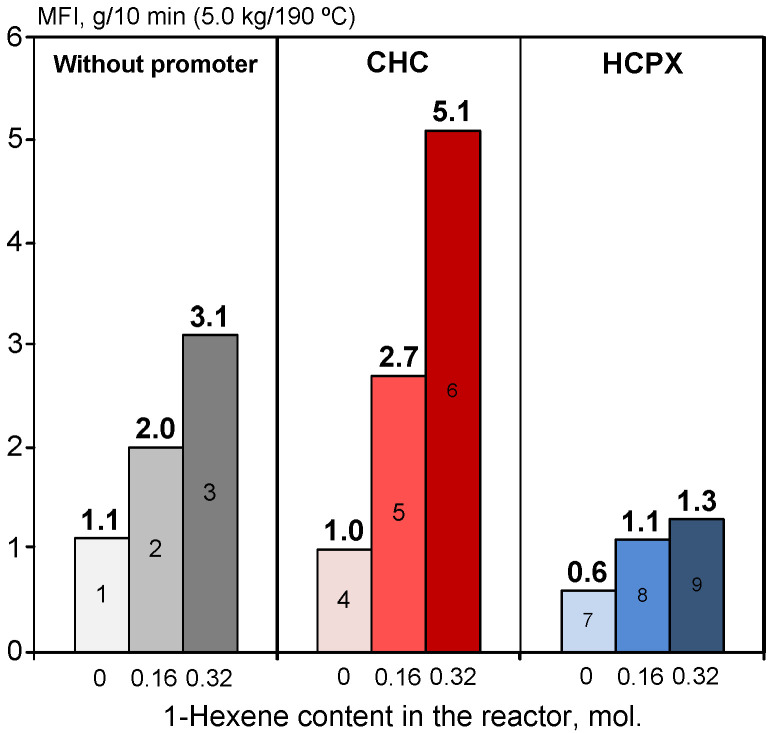
The effect of COCs on the MFI of PE samples.

Figure 4 (exp. 1, 4, and 7) demonstrates that the highest MFI was observed for the polymer produced over TMC in the absence of COC. Polyethylene synthesized in the presence of CHC had a sufficiently similar MFI, while the use of HCPX as a promoter produced the polymer with the lowest MFI value. Upon ethylene homopolymerization over the system without a COC and the TMC–CHC system, the similar MFI values agree with the sufficiently close *M*_w_ values. The polydispersity level, being somewhat higher for the sample produced in exp. 1 compared to that produced in exp. 4 (Table 2), had no significant effect on MFI. Having compared the PE samples synthesized over the TMC and TMC/HCPX systems (Figure 4; Table 2, exp. 1 and 7), one can see that the MFI decreased in a regular manner as *M*_w_ increased, while the samples were characterized by similar polydispersity.

Upon 1-hexene/ethylene copolymerization (monomer ratio, 0.57) over TMC, the MFI increased almost twofold. At higher 1-hexene contents in the system, the MFI further increased (Table 2; exp. 1–3), which agrees rather well with both the decline in molecular weight and disruption of polymer chain regularity because of incorporation of the second monomer. Similar regularities in changes in the MFI and molecular weights of the copolymers as 1-hexene content in the polymerization system was varied were also observed for the TMC–CHC and TMC–HCPX catalytic systems (Table 2, exp. 4–6 and exp. 7–9). However, the most prominent decline in molecular weight and, therefore, an increase in MFI took place for CHC used as a promoter.

For the catalyst modified with HCPX, the MFIs of both the homopolymer and the copolymers were appreciably low and changed negligibly after the second comonomer had been added and 1-hexene content had been increased, while their molecular weights were the highest (Table 2). Polydispersities of both polyethylene and copolymers were virtually independent of whether the catalytic system contained a COC or not.

**Figure 5:**
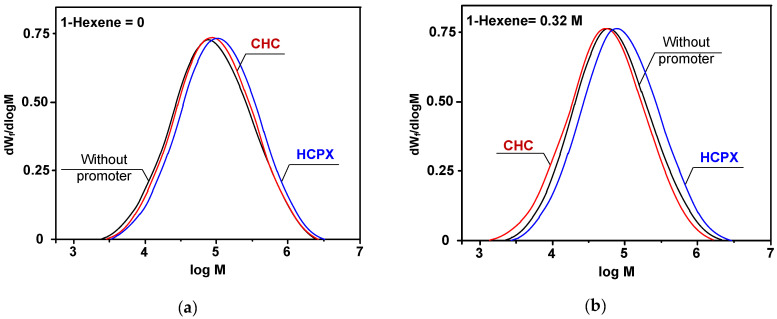
The shape of the MWD curves of PE samples: (**a**) produced upon ethylene homopolymerization in the presence of CHC and HCPX; (**b**) produced upon ethylene/1-hexene (0.32 M) copolymerization in the presence of CHC and HCPX.

Figure 5a,b show the MWD curves in the coordinates dWf/dlogM—logM for the samples of polyethylene and ethylene/1-hexene copolymers. Modification of TMC with the analyzed promoters did not significantly alter the shape of the MWD curves. The curves were unimodal for all the polymer samples.

Thus, for ethylene homopolymers (Figure 5a), modification of TMC with CHC and HCPX caused a small shift in MWD curves towards higher molecular weights, thus leading to a rise in *M*_w_. For ethylene/1-hexene (0.32 M) copolymers produced in the presence of the promoters (Figure 5b), there also were small shifts in MWD curves: towards lower molecular weights for the polymer synthesized over the catalyst modified with CHC and towards higher molecular weights for the polymer synthesized in the presence of HCPX (compared to that produced without a promoter).

Overall, the changes in molecular weight characteristics (reduction of *M*_w_ and *M*_w_/M_n_) occurring after incorporation of 1-hexene were typical of TMC and agreed with the data reported in Ref. [31].

Our findings suggest that modification of TMC with a COC insignificantly increased the number average molecular weight, other polymerization conditions being unchanged. Thus, the M_n_ of polyethylene produced over a catalyst without a COC was 39 kg/mol, while that for polyethylene produced in the presence of CHC or HCPX was 46 and 51 kg/mol, respectively. Therefore, not only do promoters increase activity of the catalytic system, but they also somewhat reduce the probability of chain termination without affecting the parameters responsible for the formation of the MWD of polyethylene (e.g., the steady state with respect to the content of active polymerization sites, or regularities of changes in concentration of polymerization active sites (AS) during the process, or the distribution of polymerization AS by their activity (if there is any), etc.).

A common feature observed for all the analyzed catalytic systems was that the addition of 1-hexene reduced the average molecular weights, being indicative of a stronger role played by chain termination processes upon copolymerization compared to homopolymerization of ethylene. The decrease in M_n_ with increasing 1-hexene content in the case of copolymerization over TMC and TMC–CHC suggested that there was a high likelihood that α-olefin was involved in polymer chain termination. This conclusion cannot be drawn in the case of using HCPX, although the probability of chain termination was also higher during copolymerization of ethylene compared to homopolymerization.

#### 2.2.2. Thermal Properties of Nascent PE Powders

Table 5 summarizes the thermal properties of the homo- and copolymer samples produced in the absence and in the presence of promoters for TMC. The DSC data for all the polyethylene samples show a single relatively broad melting peak, which indicates that thermal treatment was not accompanied by any noticeable molecular rearrangement.

Ethylene homopolymers produced over the non-modified TMC and TMC modified with a COC had similar melting points (138–139 °C) and degrees of crystallinity (61–64%) (Table 5, exp. 1, 4, and 7), which are typical of HDPE. The small difference in the melting points and degrees of crystallinity of PE samples was probably related to the differences in molecular weight.

Therefore, the promoters analyzed in this study had no effect on the thermal properties of polyethylene formed over TMC.

The copolymers produced over all the studied catalytic systems had lower melting points (131–132 °C) and degrees of crystallinity (40–51%) (Table 5, exp. 2, 5, and 8) compared to those of homopolymers, which is quite predictable. The minimal degree of crystallinity (40%) was observed for the copolymer with the maximum comonomer content (Table 2, exp. 2) that was produced over the non-modified TMC. The effect of COC on the degree of crystallinity of the polymers was related to 1-hexene content in the copolymer.

### 2.3. The Mechanistic Aspects of TMC Modification with Chlorinated Organic Discussion Compounds and Variation in Its Copolymerization Ability

The mode of action of the activating effect of promoters carrying a labile chlorine atom on titanium–magnesium Ziegler–Natta catalysts (TiCl_4_·MgCl_2_/AlR_3_) upon ethylene polymerization remains a matter of debate. Several hypotheses have been put forward:

1. Regeneration (reactivation) of catalytic sites with low activity as a result of oxidation of low-valent titanium [4,8,32].
2. The lower reducing ability of organoaluminum compounds as alkyl groups are substituted with chlorine atoms in the reactions between AlR_3_ and RCl, as well as the lower rates of reductive processes in the catalytic system [8]; it can change the Ti^2+^/Ti^3+^ ratio in active sites.
3. Formation of a small amount of Lewis acids upon interaction between AlR_3_ and RCl (AlRCl_2_, AlCl_3_) [8]; Lewis acids can bind products that are formed during polymerization (AlR_2_H, AlR_2_Cl, etc.) and block active sites, thus increasing the concentration of chain growth sites [33].
4. Halogenated hydrocarbons can be weakly coordinated to the titanium active site, thus changing its reactivity [14].

Modifying TMC with a COC affects the redistribution of Ti^2+^/Ti^3+^ within the active component of the catalyst, which is formed after the interaction between TMC and an organoaluminum cocatalyst (TIBA). Therefore, the maximum percentage of Ti^2+^ is formed in the non-modified TMC; the percentage of Ti^2+^ decreases, while the percentage of Ti^3+^ increases in TMC–CHC. The minimal percentage of Ti^2+^ (and the maximal percentage of Ti^3+^) is observed for the TMC–HCPX catalyst. The results of DFT studies confirmed [4,32] that, for chlorine-rich promoters, oxidation (reactivation) of the Ti^2+^ site to the Ti^3+^ site was thermodynamically more favorable.

The increasing activity observed upon the modification of TMC with a COC can also be potentially attributed to a higher yield of active sites in Ti^3+^ compounds, which are predominant in these systems. The low-valent Ti^2+^ compounds, whose content is probably higher in the non-modified TMC, are more likely to form complexes with the products formed during polymerization (e.g., with aluminum hydrides produced in the presence of hydrogen) [33]. This may block some of the active sites in these catalysts and reduce their activity.

The plausible reason for the effect of COCs on the copolymerization ability of TMC and the molecular weight of the resulting copolymer is as follows. The TMC modified with HCPX yields copolymers with a lower 1-hexene content. Modification of TMC with HCPX is probably caused by changes in the ligand environment of titanium.

Furthermore, earlier data [33] suggest that TMCs containing Ti^2+^ in their active site are characterized by a higher probability of α-olefin incorporation into the polymer chain upon ethylene/α-olefin copolymerization than the TMC/COC systems. In this study, the copolymerization constant of the non-modified TMC (r_1_ = 75) is similar to the data (r_1_ = 65) obtained in ref. [33]. If a catalytic system contains no COC, the percentage of active sites containing Ti^2+^ is higher compared to the systems modified with a promoter. The r_1_ values for the Ti^3+^-containing catalysts range from 100 to 114 [33], which is close to the data obtained in this study for the TMC modified with CHC. Therefore, the r_1_ value for the non-modified TMC is lower, while the α-olefin content is higher than the respective values for the TMC/COC systems, where the active sites predominantly contain Ti^3+^ compounds.

**Scheme 1:**
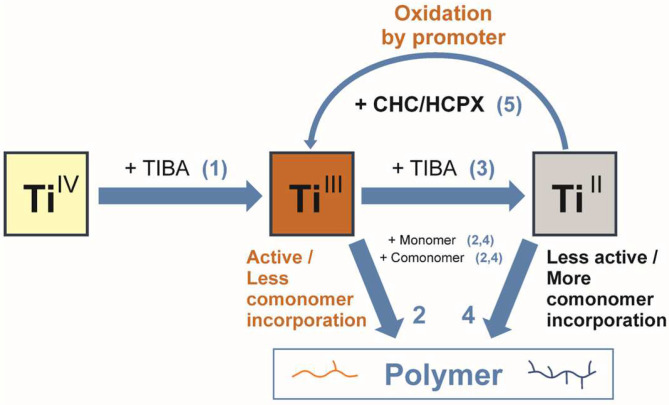
The simplified reaction pathways involved in ethylene/1-hexane copolymerization over the titanium–magnesium catalyst system and chlorocyclohexane/hexachloro-p-xylene.

Ethylene/1-hexene copolymerization over TMC/TIBA/COC can be shown using the following simplified Scheme 1:

In the absence of chlorinated organic compounds, the process occurs in the following directions: (1) formation of active sites (+TIBA); (2) copolymerization over Ti^3+^ (monomers and comonomers); (3) further reduction of titanium (+TIBA); and (4) copolymerization over Ti^2+^ (monomers and comonomers). After a COC was added, a new process appears: (5) the oxidation of Ti^2+^ and the returning of a certain percentage of titanium to the formation of new active sites with a higher oxidation number Ti^3+^ (+CHC/HCPX). Furthermore, a new chlorinated organic component (CHC or HCPX) should be added to direction 1, since the higher rate of the process is associated not only with oxidation Ti^2+^ → Ti^3+^, but also with the fact that the copolymerization constants (Table 4) and sensitivity to hydrogen (Figure 4, Table 5) change differently, which indicates that CHC/HCPX is very likely to be involved in the formation of copolymerization sites.

Speaking about the sensitivity to hydrogen for the TMC modified with HCPX, it should not be ruled out [34] that, upon coordination on an active site, a COC containing the benzene ring can simultaneously (a) activate the process due to oxidation by chlorine and (b) form steric hindrance upon interaction with 1-hexene and hydrogen.

## 3. Materials and Methods

### 3.1. Reagents and Catalysts

The titanium–magnesium catalyst (TMC) was prepared according to the procedure described in Ref. [35]. The TMC used in this study contained 5.4 wt.% titanium and 14 wt.% magnesium; it was characterized by narrow particle size distribution (SPAN = 0.68); the average particle size was 38 µm.

**Scheme 2:**
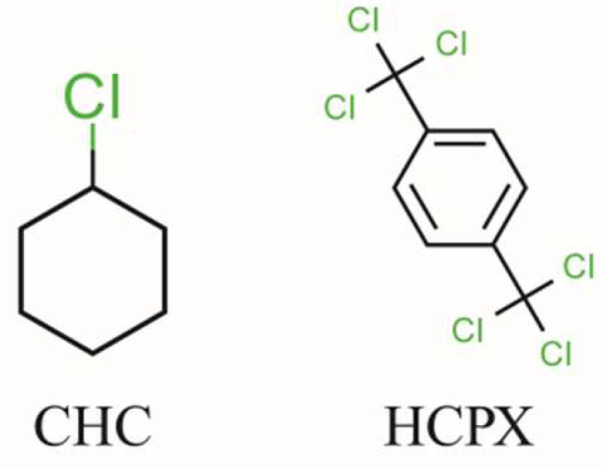
The structures of CHC and HCPX.

Hexachloro-p-xylene (HCPX; 1,4-bis(trichloromethyl)benzene C8H4Cl6; main substance content ≥ 98%, CAS No. 68-36-0) and chlorocyclohexane (CHC; 1-chlorocyclohexane C6H11Cl; main substance content ≥ 98%, CAS No. 542-18-7), were used as chlorinated organic compounds (Scheme 2).

HCPX and CHC were added to the catalyst slurry in heptane at room temperature at a COC/Ti molar ratio = 1. The slurry was exposed to room temperature under stirring for 40–60 min. To perform ethylene polymerization, the cured mixture of TMC slurry and COC was dosed into spherical glass ampoules with long “tails” in an argon atmosphere and sealed off.

Polymer-grade ethylene (PJSC “Kazanorgsintez”) was subjected to additional purification using columns packed with reduced manganese catalyst and molecular sieves to remove oxygen and moisture. The purified monomer contained ≤0.0005 wt.% oxygen and ≤0.001 wt.% water. Heptane (EKOS-1) and 1-hexene (PJSC “Nizhnekamskneftekhim”) were dehydrated above molecular sieves. The purified compounds contained ≤0.001 wt.% moisture. High-purity argon gas of grade 5.5 was additionally purified over a reduced nickel–chromium catalyst to remove oxygen and molecular sieves were used to remove moisture. Oxygen content in argon was ≤0.0001 wt.%; water content was ≤0.0003 wt.%. Triisobutylaluminum (TIBA) produced by PJSC “Nizhnekamskneftekhim” was used without additional purification as a solution in heptane (0.2 mol/L).

### 3.2. Chemical Analysis of the Catalysts

After being treated with COC, the TMC samples were dried (without washing) and analyzed to determine the titanium and magnesium contents. The titanium and magnesium contents were measured by inductively coupled plasma atomic emission spectrometry on an Optima 4300 DV spectrometer.

### 3.3. Determining Particle Size Distribution and the Average Catalyst Particle Size

The particle size distribution (SPAN) and the average particle size (d50) of the catalysts were determined by laser diffraction (Malvern) on a Mastersizer 2000 particle size analyzer. The SPAN value was determined as (d90–d10)/d50. The particle sizes d10, d50 and d90 corresponded to 10, 50, and 90 wt.% catalyst particles.

### 3.4. Slurry-Phase Polymerization

Ethylene polymerization and ethylene/1-hexene copolymerization were carried out in a 0.85 L stainless steel autoclave equipped with a magnetic stirrer, a jacket for maintaining desired temperature, and a device for breaking ampoules containing the catalyst. Air was evacuated from the autoclave at 80 °C during 1.5 h. After cooling down to 50 °C, the calculated amounts of the solvent (250 mL) and TIBA as solution in heptane ([Al] = 4.8 mmol/L) were added into the autoclave. In the case of copolymerization, 5–10 mL of 1-hexene was also added. The duration of polymerization and copolymerization reactions was 30 min. Parameters such as the total rate of the process and monomer contents in the initial mixture and the resulting copolymer were assessed.

The initial pressure in the autoclave at 80 °C was maintained at ~0.3 atm, and the calculated amounts of H_2_ (1 atm) and ethylene (4 atm) were added. The instant when the ampoule with the catalyst was broken was considered the instant of polymerization initiation. TMC concentration in the reactor was ~0.03 g/L. Polymerization was performed at 80 °C, constant ethylene pressure, and a constant hydrogen/ethylene molar ratio in the gas phase. The reaction rate was calculated according to the readings of flow meter measuring the flow rate of ethylene supplied into the reactor during polymerization. The flow meter readings were exported to a PC with a certain time interval for calculating and outputting the polymerization rate data in graphical and tabular formats. Once the reaction had been completed, the autoclave was cooled down to room temperature; ethylene was released from the reactor, and the PE slurry was unloaded into a metal tray. After the slurry had been allowed to settle down, the transparent liquid phase was decanted, the polymer was dried to constant weight, and the weight of PE was determined.

Activity of TMC (*W_G_*) was calculated using the formulas:

*W_G_* = Y × 100/m (kg PE/g cat × h),

where Y is the yield of the polymer per hour; m is the weight of TMC added into the reactor (g); *W_Ti_* is the yield of the polymer per unit mass of titanium in the polymerization system:

*W_Ti_* = *W_G_* × 100/(%M × P) (kg PE/g M × h × atm),

where %M is the percentage content of transition metal in the catalyst; P is monomer pressure, atm.

### 3.5. Determining the Copolymerization Constants

The constants of ethylene/1-hexene copolymerization over the TMC/TIBA, TMC/TIBA/CHC, and TMC/TIBA/HCPX catalytic systems were calculated using the methods proposed in Refs. [36,37], which can be used at low α-olefin contents in the copolymer. The constants r_1_ and r_2_ were determined according to equations:

(Cα/C_2_H_4_)_polymer_ = 1/r_1_ × [Cα]/[C_2_H_4_],

where (Cα/C_2_H_4_) are the molar ratios between α-olefin and ethylene in the copolymer; [Cα] and [C_2_H_4_] are the α-olefin and ethylene concentrations in the reaction medium.

[F/f] × (f − 1) = r_1_ × [F/f] − r_2_

where F = [M_1_]/[M_2_] and f = [m_1_]/[m_2_]; m_1_ and m_2_ are the monomeric units 1 and 2 in the copolymer; [M_1_] and [M_2_] are their initial concentrations in the original mixture; r_1_ is the copolymerization constant, which is characterized by the ratio between the rate constant of ethylene attachment to the ethylene monomer of propagating chain (k_11_) and the rate constant of olefin attachment to the ethylene monomer of propagating chain (k_12_).

The experimental data were used to plot the diagram y = y (x), where y = F (f − 1)/f and x = F2/f. Each experiment (i.e., the pair of F and f values) yielded a point on this diagram, while a series of experiments yielded a straight line. The slope of the straight line corresponded to the r_1_ value, while the intercept on the Y axis corresponded to the r_2_ value with the sign reversed.

### 3.6. Analyzing Properties of the Polymers

The melt flow indices (MFI) of the polymers were determined at 190 °C at loads of 5 kg according to the ASTM D1238 test method.

The average molecular weights (*M*_w_, M_n_) and the molecular weight distribution of the polymers were determined on a PL-220 HPLC system equipped with a set of PL Gel Olexis columns, as well as refractometric and differential viscometric sensors at 160 °C; 1,2,4-trichlorobenzene (TCB) was used as an eluent (elution rate, 1 cm^3^/min).

The content of CH_3_ groups (absorption band at 1378 cm^−1^) in the polymer was determined using an 8400S FTIR spectrometer (Shimadzu) [38].

The melting point of the polymer samples was measured by differential scanning calorimetry (DSC) on a 204 F1 differential scanning calorimeter (Netzsch, Germany) in an argon atmosphere (flow rate, 30 mL/min) in closed 25 µL aluminum crucibles according to the ASTM D3418-82 and ASTM D3417-83 procedures. The instrument was calibrated with the standard samples, indium and zinc (Aldrich). The DSC curves of the sample were recorded in the melting–crystallization–melting mode in the temperature range of 25–180 °C at a rate of 10 °C/min. The melting point and the enthalpy of fusion were measured according to the data for the second melting.

The degree of crystallinity of the samples (X) was determined using the formula:

X = ΔHsample × 100%/ΔH100%PE,(1)

where ΔHsample is the enthalpy of the sample; ΔH100%PE = 290 J/g is the enthalpy of the fusion of polyethylene with 100% degree of crystallinity.

## 4. Conclusions

The results of studying ethylene polymerization and ethylene/1-hexene copolymerization over the TiCl_4_·MgCl_2_/TIBA catalytic system in the presence of mono- and multichlorinated organic compounds revealed that chlorocyclohexane and hexachloro-p-xylene allow one to increase the activity of TMC upon homopolymerization two- to four-fold and, upon copolymerization, 1.2–2.0-fold. In other words, these compounds are efficient promoters of these processes. The nature of COC was found to affect the MFI and molecular characteristics of PE, while differences in these parameters become more obvious when the catalytic system is modified with 1-hexene. As 1-hexene content was increased, the M_n_ declined upon copolymerization over TMC and TMC–CHC, but this conclusion cannot be drawn for HCPX.

The constants of ethylene/1-hexene copolymerization over non-modified TMC and TMC in the presence of CHC and HCPX have been calculated for the first time. The addition of CHC and HCPX to the system based on titanium–magnesium catalyst increased the copolymerization constant r_1_ and reduced r_2_ at r_1_ × r_2_ = 1; therefore, a smaller amount of 1-hexene was incorporated into the copolymer.

From the perspective of the mechanistic aspects of the effect of chlorine-containing promoters, the observed changes in the copolymer structure are potentially caused by the fact that the modified catalyst produces a polymer with a higher crystallinity. It can be easily explained as follows: when the pristine TMC is modified with a COC, the concentration of less active sites with an increased copolymerization ability (Ti^2+^) is shifted towards more active sites with a lower copolymerization ability (Ti^3+^), and the resulting polymer is characterized by a high yield and crystallinity. As for sensitivity to hydrogen during copolymerization, it has been suggested that active sites in the TMC modified with CHC are more labile (having a higher likelihood of chain termination), while in the case of TMC modified with HCPX, active sites with higher stability (and lower likelihood of chain termination) are formed. This feature can also be attributed to the nature of hydrocarbon in COC: the cyclic ring for CHC and the aromatic ring for HCPX. They both seem to be able to ensure certain steric hindrance for the active site due to coordination and reduce the incorporation of 1-hexene (low r_2_), while in the case of HCPX, it is the interaction with hydrogen (the low MFI and increased *M*_w_).

The increased efficiency of titanium–magnesium Ziegler–Natta catalysts revealed in this study, and the modification of the end-use properties of copolymers by using chlorocyclohexane and hexachloro-p-xylene in the catalytic system, are of significant interest for the industrial-scale production of novel polyolefin brands. The application of these promoters to the synthesis of ethylene homopolymers is undoubtedly a preferable and more interesting solution for increasing the polymer yield.

## Figures and Tables

**Table 1:** The effect of COCs on activity of TMC during ethylene homopolymerization.

Exp. No.	Type of COC	COC/Ti Molar Ratio	Yield (*W_G_*), kg PE/g cat∙h	Activity (*W_Ti_*), kg PE/g Ti∙h
1	Without promoter	-	1.8	33.3
4	CHC	1:1	6.7	136.7
7	HCPX	1:1	3.8	122.6

Polymerization conditions: heptane (250 mL), [cat] = 0.020–0.025 g/L; 80 °C; P_C2H4_ = 4 atm; P_H2_ = 1 atm; [TIBA] = 4.8 mmol/L; 30 min.

**Table 2:** The effect of COCs on activity of TMC during ethylene homopolymerization and ethylene/1-hexene copolymerization.

Exp. No.	Name of Promoter	COC/Ti (Molar Ratio)	[1-Hexene], M	[C_6_H_12_]/[C_2_H_4_] (mol/mol)	*W_G_*, kg PE/g cat∙h	*W_Ti_*, kg PE/g Ti∙h
1	Without promoter	-	0	0	1.8	33.3
2		-	0.16	0.57	4.3	79.6
3		-	0.32	1.14	3.2	59.3
4	CHC	1:1	0	0	6.7	136.7
5		1:1	0.16	0.57	7.0	142.9
6		1:1	0.32	1.14	6.5	132.7
7	HCPX	1:1	0	0	3.8	122.6
8		1:1	0.16	0.57	5.2	167.7
9		1:1	0.32	1.14	4.0	129.0

Polymerization conditions: heptane (250 mL), [cat] = 0.020–0.025 g/L; 80 °C; P_C2H4_ = 4 atm; P_H2_ = 1 atm; [TIBA] = 4.8 mmol/L; 30 min.

**Table 3:** The monomer ratio in the initial mixture and in the resulting copolymer.

Exp. No.	Name of Promoter	[1-hexene], M	Initial [C_6_H_12_]/[C_2_H_4_] (mol/mol)	[C_6_H_12_] mol.%. Polymer	Density, g/cm^3^
1	Without promoter	0	0	-	0.949
2		0.16	0.57	0.96	0.936
3		0.32	1.14	1.2	0.933
4	CHC	0	0	-	0.949
5		0.16	0.57	0.58	0.938
6		0.32	1.14	1.12	0.935
7	HCPX	0	0	-	0.949
8		0.16	0.57	0.26	0.942
9		0.32	1.14	0.56	0.938

Polymerization conditions: heptane (250 mL), [cat] = 0.020–0.025 g/L; 80 °C; P_C2H4_ = 4 atm; P_H2_ = 1 atm; [TIBA] = 4.8 mmol/L; 30 min.

**Table 4:** The constants of ethylene/1-hexene copolymerization over TMC in the presence of COC in the catalyst. The conditions are listed in Table 1.

Exp. No.	Catalytic System	The Constants of Ethylene (Monomer 1)/ Hexene 1 (Monomer 2) Copolymerization		r_1_ × r_2_
		r_1_	r_2_	
1	TMC–TIBA	75	0.013	1.0
2	TMC–TIBA–CHC	101	0.010	1.0
3	TMC–TIBA–HCPX	202	0.005	1.0

**Table 5:** The molecular and thermal properties of polyethylene samples produced over TMC/TIBA catalyst (without a promoter and in the presence of CHC and HCPX).

Exp. No.	Name of Promoter	[1-Hexene], M	*M*_w_, kg/mol	M_n_, kg/mol	*M*_w_/M_n_	T_m_, °C	Crystallinity, %
1	Without promoter	0	170	39.5	4.3	137.9	62
2		0.16	145	37.0	3.9	130.3	55
3		0.32	120	31.5	3.8	130.8	40
4	CHC	0	180	46	3.9	137.5	64
5		0.16	140	37	3.8	133.7	54
6		0.32	100	26	3.8	131.0	48
7	HCPX	0	200	51	3.9	138.6	61
8		0.16	165	44	3.8	134.5	54
9		0.32	160	42	3.8	132.3	51

## Data Availability

Data are available at request from the authors.
